# Myxomaviral Anti-Inflammatory Serpin Reduces Myeloid-Derived Suppressor Cells and Human Pancreatic Cancer Cell Growth in Mice

**DOI:** 10.4172/1948-5956.1000219

**Published:** 2013-08-19

**Authors:** Donghang Zheng, Hao Chen, Mee Y Bartee, Jennifer Williams, Jennifer A Davids, David A Lomas, Grant McFadden, Alexandra R Lucas

**Affiliations:** 1 Department of Medicine, University of Florida, Gainesville, FL, USA; 2 Department of Molecular Genetics and Microbiology, University of Florida, Gainesville, FL, USA; 3 University of Cambridge, Cambridge, UK

**Keywords:** Serpin, Serp-1, Pancreatic cancer, Myeloid-derived suppressor cell, Tumor-associated macrophage

## Abstract

Modification of the tumor microenvironment by inflammatory cells represents a newly recognized driving force in cancer with critical roles in tumor invasion, growth, angiogenesis, and metastasis. Increased thrombolytic cascade serine proteases, specifically urokinase-type plasminogen activator and its receptor, correlate with inflammatory cell migration, pancreatic cancer growth, invasion and unfavorable outcomes. Inflammation in pancreatic cancer is linked with myeloid-derived suppressor cell (MDSC) activity and cancer progression. Myxomavirus is a complex DNA virus encoding highly potent immune modulators. Serp-1 and M-T7 are two such secreted anti-inflammatory myxomaviral proteins. Serp-1 inhibits uPA, plasmin and coagulation factor X while M-T7 inhibits C, CC, and CXC chemokines. We have explored the potential use of these viral proteins for treatment of a range of human cancer isolates engrafted in severe combined immunodeficient (SCID) mice. Engrafted tumors were treated with either Serp-1, neuroserpin, a related mammalian serpin that inhibits thrombolytic proteases, or M-T7. Serp-1 and neuroserpin inhibited growth of the pancreatic cancer cell line Hs766t (P=0.03 and P=0.01, respectively) at 4 weeks after implantation. Serp-1 also inhibited growth of a second pancreatic cancer cell line MIA PaCa-2 in mice (P=0.02). Growth of the human breast cancer line MDA231 was not inhibited by Serp-1. M-T7, in contrast, did not alter growth of any of the cancer cell lines tested after implant into SCID mice. Serpin inhibition of pancreatic tumor growth was associated with a significant decrease in splenocyte MDSC counts by flow cytometry (P=0.009), without detected change in other splenocyte subpopulations. Serp-1 and NSP treatment also significantly reduced macrophage infiltration in tumors (P=0.001). In summary two anti-inflammatory serpins reduced inflammatory macrophage invasion and pancreatic tumor cell growth, suggesting potential therapeutic efficacy.

## Introduction

It is now recognized that chronic inflammation is associated with cancer. More recent evidence also supports a central role for innate immune responses and more specifically for serine proteases, chemokines, and their receptors in every stage of cancer development including initial cell invasion, tumor cell proliferation and angiogenesis, as well as metastasis [1]. Under conditions of chronic inflammation, reactive oxygen or nitrogen species produced by inflammatory cells may initiate oncogenesis by causing DNA damage in adjacent cells. Inflammatory cells also produce mediators such as proteases, cytokines, chemokines, and growth factors, which promote cancer cell growth, migration, and angiogenesis. For example, accumulation of tumor-associated macrophages is associated with poor prognosis in selected cancers, including pancreatic cancer [2–4]. Some cancers also induce the presence of increased numbers of myeloid-derived suppressor cells (MDSCs) that block innate and acquired immune responses mounted by the host against the invading cancers [5]. Among mediators that drive inflammation are the serine proteases in the thrombotic and thrombolytic cascades, in particular the urokinase-type plasminogen activator (uPA) and its receptor (uPAR). uPA activates matrix degrading enzymes, allowing cell invasion, as well as growth factors, such as transforming growth factor beta (TGFβ) that can stimulate tumor growth. Elevated levels of both uPA and uPAR are detected in association with pancreatic cancer, breast cancer and other cancers [6]. Based upon the fact that inflammation is an important promoting factor for cancer development in many cases, therapeutics targeting inflammatory pathways and specifically the uPA/uPAR complex or chemokines and their receptors may represent a new strategy for cancer treatment.

Myxomavirus uses highly effective immuno-suppressive proteins to circumvent host defenses against viral infections, with inhibitory activity for host responses often achieved at femtomolar quantities for these secreted anti-inflammatory agents. One of the most potent of these proteins is Serp-1, a serine protease inhibitor (serpin) that inhibits urokinase-type and tissue-type plasminogen activators (uPA and tPA), plasmin, coagulation factor X (FX) as well as factor II (FII, thrombin) under select conditions in the presence of the glycosaminoglycan (GAG), heparin. Serp-1 is a critical virulence factor for myxomavirus, functioning as an immunosuppressor in infected host European rabbits [7]. The immunomodulatory activity of purified Serp-1 has been demonstrated in multiple animal disease models by our lab and others [8]. In vascular injury and inflammation models, Serp-1 decreases inflammatory cell infiltration and intimal plaque formation in the injured arteries [9–12]. Serp-1 also inhibits angiogenesis in a chicken chorioallantoic membrane (CAM) model [13]. Similarly, neuroserpin (NSP), a mammalian serpin that inhibits uPA and tPA, has anti-inflammatory activity in an aortic allograft vasculopathy model [14]. In a recent clinical trial, Serp-1 has also been demonstrated to reduce markers for myocardial damage in patients with acute coronary syndromes undergoing percutaneous coronary intervention with coronary stent implant, with minimal side effects [15].

Another class of viral anti-inflammatory proteins, the chemokine-modulating proteins (CMPs), functions through interruption of chemokine interactions with cells in the innate immune response system. These CMP can block chemokine activity either in a direct manner through interruption of the chemokine - chemokine receptor interactions or indirectly through interruption of chemokine - GAG interactions. M-T7 is a myxomaviral CMP, which binds to the GAG-binding domains of C, CC and CXC chemokines, disrupting formation of the chemokine gradients that are essential for immune cell migration. Anti-inflammatory activity of M-T7 has also been demonstrated in animal models of balloon angioplasty, aortic transplantation, and renal transplantation [16–18].

We have postulated that the viral anti-inflammatory serpins, such as Serp-1, and CMPs such as M-T7 will block tumor associated leukocyte activity and tumor progression. With these studies we have explored the potential of anti-inflammatory viral serpins and chemokine modulators in cancer treatment, examining the efficacy of Serp-1, neuroserpin (a related mammalian serpin), and M-T7 for inhibition of selected human tumor cell lines in immunodeficient NOD/SCID mice.

## Materials and Methods

### Proteins and antibodies

Purified Serp-1 protein was kindly provided by Viron Therapeutics, Inc. (London, ON. Canada). His-tagged neuroserpin protein was expressed in BL21 (DE3) pLysS cells (Invitrogen, Carlsbad, CA, USA), and purified using cobalt NTA resin (Sigma-Aldrich, St. Louis, MO, USA), as previously described [14]. M-T7 was produced using baculovirus-mediated expression system (Invitrogen, Carlsbad, CA, USA), as previously described [19]. Mouse uPA was purchased from HYPHEN BioMed (Neuville sur Oise, France).

Anti-Ki67 monoclonal antibody (ab16667) and anti-F4/80 monoclonal antibody (ab111101) were purchased from Abcam Inc. (Cambridge, MA, USA) for immunohistochemistry. PerCP-Cy5.5 anti-mouse Ly-6G/Ly-6C (Gr-1) antibody (cat. 108428) was purchased from BioLegend Inc. (San Diego, CA, USA). PerCP-Cy5.5 conjugated anti-mouse CD3 antibody (cat. 45-0031-80) and APC-eFluor 780 conjugated anti-mouse CD11b antibodies (cat. 47-0112-80) were purchased from eBioscience Inc. (San Diego, CA, USA).

### Animal studies and cancer cell transplantation

All animal protocols and surgical procedures conform to national and local guidelines and were approved by the University of Florida Local Animal Care and Research Committee (IACUC). All animals receive care in compliance with the Principles of Laboratory Animal Care, and the approved experimental protocol. NOD.CB17-Prkdc (NOD/SCID) mice (stock number 001303) were obtained from the Jackson Lab (Sacramento, CA, USA). All animals were held under standard conditions at constant temperature, humidity, and light/dark cycles, fed with a standard diet, and had free access to tap water.

Human pancreatic cancer cell lines Hs766t (ATCC HTB-134) and MIA PaCa-2 (ATCC CRL-1420), human breast cancer cell line MDA231 (ATCC HTB-26), human acute myelogenous leukemia cell line KG-1 (ATCC CCL-246), and human hepatocarcinoma cell line Huh7.5 were cultured in monolayer with DMEM supplemented with 10% fetal bovine serum (FBS).

5×10^6^ cells were injected to the flank of each NOD/SCID mouse subcutaneously. Mice transplanted with Hs766t cells and MDA231 were treated each day with either saline, neuroserpin (100 ng/gram body weight), Serp-1 (100 ng/gram body weight), or M-T7 (100 ng/gram body weight) through intra-peritoneal (i.p.) injection for 2 weeks. Mice transplanted with MIA PaCa-2 cells were treated with saline or Serp-1 (100 ng/gram body weight) through i.p. injection for 2 weeks (Table 1). Four weeks after transplantation, tumors were excised and weighed. Part of each isolated tumor harvested from mice at 4 weeks follow up were preserved in 10% formalin for histological study. Spleens were also harvested for flow cytometry study. For the time course study analysis of pancreatic cancer cell growth was performed in NOD/SCID mice transplanted with Hs766t cells and treated each day with either saline or Serp-1 (100 ng/gram body weight) by i.p. injection throughout the study. Animals were sacrificed at days 14, day 21, and day 28 post transplantation and tumors were weighed. There were three mice in each group (n=3 for saline group, n=3 for Serp-1group) at each time point.

For uPA augmentation study, 5 × 10^6^ Hs766t cells were suspended in 1000u of uPA (in 100 μl of distilled water) and were transplanted to the back of a NOD/SCID mouse subcutaneously, followed by i.p. injection of saline or Serp-1 (100 ng/gram body weight) for 2 weeks. Animals were sacrificed 4 weeks after transplantation and tumor tissues and the spleens were collected for analysis.

### *In vitro* cell proliferation assay

Hs766t cells were cultured in a 96-well plate initiating with 3000 cells/well. Cells were treated with Serp-1 at a final concentration of 1.0, 2.0, or 4.0 μg/ml or saline. All treatments are in triplicate. Cell proliferation was assessed after 24, 48, 72 and 96 hours by MTT (3-(4, 5-Dimethylthiazol-2-yl)-2,5-diphenyltetrazolium bromide) assay using CellTiter 96 Non-Radioactive Cell Proliferation Assay Kit (Promega, Madison, WI, USA).

### Immunohistochemistry

EXPOSE rabbit specific HRP/DAB detection IHC kit (abcam, ab80437) was used for immunostaining to detect anti-Ki67 for proliferation and anti-F4/80 for tissue macrophage as previously described [16,17,19]. In brief, de-paraffinized tissue sections were blocked with hydrogen peroxide blocking agent for 30 minutes. Antigen recovery was performed by boiling tissue sections in diluted antigen retrieval buffer (abcam, ab64236) for 20 minutes followed by cooling in room temperature for 20 minutes. After protein block, the tissue sections were incubated with anti-Ki67 antibody or anti-F4/80 antibody overnight at 4°C. After three washes in PBS, the sections were incubated with HRP conjugate for 30 minutes at room temperature followed by three washes in PBS. Staining was developed by adding DAB substrate solution to the tissue sections. An Olympus BX51 microscopy coupled with Olympus DP71 digital camera was used for examining and generating the images of the tissue sections.

### Flow cytometry

Splenocytes were isolated by gently mashing the spleen with the rubber tip of a syringe plunger through cell strainer to a petri dish. After lysis of red blood cells and two washes with PBS, single cell suspensions in PBS were stained with mixtures of antibodies to CD11b and Ly-6G/Ly-6C (Gr-1). Cell samples were also stained with isotype control antibodies. Flow cytometry was performed with a CyAn ADP Analyzer (Dako, Ft Collins, CO) as previously described [18]. The data was analyzed using Gatelogic software (eBioscience, San Diego, CA).

### Statistical analysis

Data from all studies was analyzed using Analysis of variance (ANOVA) together with Fisher’s PLSD, and Student’s unpaired T-test using Stat View software from SAS institute Inc. (Cary, NC, USA).

## Results

### Serpins inhibited pancreatic cancer cell growth, but not growth of breast cancer cells in NOD/SCID mice

**Figure 1:**
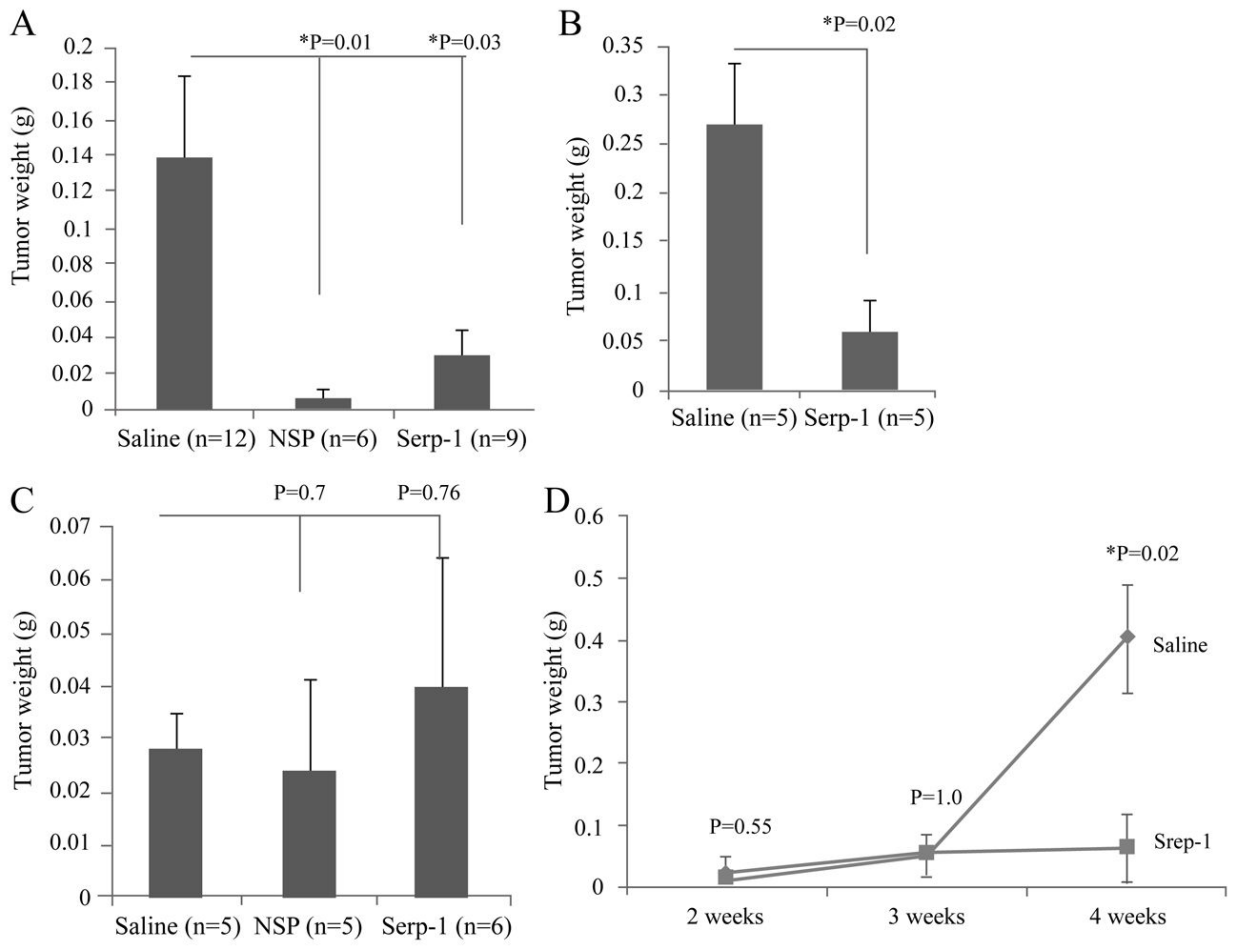
Serpins inhibit pancreatic cancer cell growth but not breast cancer growth in NOD/SCID mice NOD/SCID mice transplanted with Hs766t (n =27 total), MIA PaCa-2 (n =10 total) or MDA231 cells (n =16 total) were treated with saline, Serp-1, or neuroserpin for 2 weeks. Four weeks after transplantation, tumors excised from each group were weighed. (A) Growth of Hs766t xenografts was inhibited in mice receiving neuroserpin (n =6, P= 0.01) or Serp-1 (n =9, P= 0.03) treatment, when compared to saline (n =12). (B) Serp-1 treatment (n=5) also inhibited growth of another pancreatic cancer cell line, MIA PaCa-2 in NOD/SCID mice when compared to saline treatment (n =5, P ≤ 0.02). (C) Growth of the breast cancer cell isolate, MDA231 cells in NOD/SCID mice was not inhibited by neuroserpin (n=5, P= 0.76) or Serp-1 (n=6, P= 0.70). (D) In the time course study, NOD/SCID mice transplanted with Hs766t cells were treated each day with saline or Serp-1 throughout the 4 week experiment. Tumor suppressive effects of Serp-1 were evident after three weeks of treatment (P < 0.02). * indicates significance of P ≤ 0.05.

We began with an assessment of the capacity of the antiinflammatory serpins, Serp-1 and NSP, to modify growth of a range of human cancer tumor cell lines when implanted in NOD/SCID mice (Table 1). Serp-1 and NSP both inhibit uPA and tPA whereas Serp-1 also inhibits FII and FX while NSP does not. Serp-1 and NSP treatment for 14 days starting from day one, significantly reduced growth of the pancreatic cancer cell line Hs766t after transplant into NOD/SCID mice (P<0.03 and P< 0.01, respectively) (Figure 1A). Anti-tumor activity after Serp-1 treatment was also seen with MIA PaCa-2 pancreatic cancer cells (P ≤ 0.02, Figure 1B). In contrast, growth of the breast cancer cell line MDA231 after transplant into NOD/SCID mice was not altered by serpin treatment (P=0.76 and 0.70, respectively for NSP and Serp-1; Figure 1C).

A time course study further confirmed inhibition of Hs766t pancreatic cancer growth by 4-weeks with Serp-1 treatment (P< 0.02), but without significant inhibition at earlier follow-up times (Figure 1D; P<0.02 at 4 weeks). As Serp-1 inhibits both thrombolytic serine proteases tPA and uPA and also thrombotic proteases FX and FII, whereas NSP inhibits only tPA and uPA, we have posited that the majority of the anti-tumor activity is dependent upon the inhibition of the thrombolytic proteases tPA and uPA.

### M-T7 did not suppress growth of cancer cell lines in NOD/SCID mice

The myxoma virus-derived chemokine modulating protein (CMP), M-T7, also has proven anti-inflammatory activity in animal models of balloon angioplasty injury and aortic transplant, through interruption of chemokine:GAG interactions [16,18]. We have therefore examined the capacity of M-T7 to alter growth of cancer cell isolates after implant into NOD/SCID mice. To assess the anti-tumor activity of this class of anti-inflammatory chemokine modulator, M-T7 was also used to treat the NOD/SCID mice after implant with the human pancreatic cancer cell line Hs766t, the human breast cancer cell line MDA-231, the human myelogenous leukemia cell line KG-1, and the human hepatocarcinoma cell line Huh7.5.

**Figure 2:**
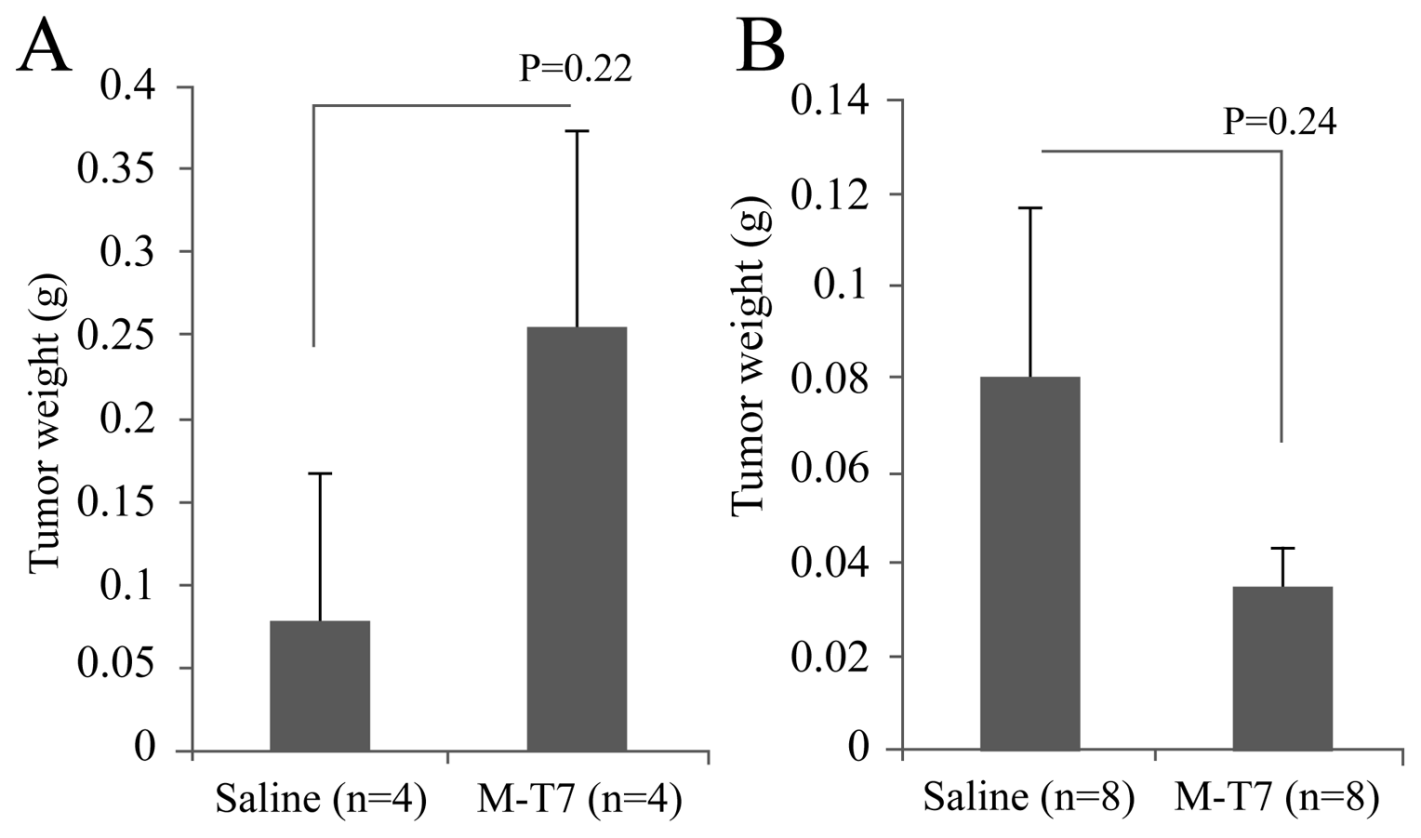
M-T7 did not inhibit growth of cancer cell lines in NOD/SCID mice NOD/SCID mice transplanted with Hs766t or MDA231 were treated with M-T7 for 2 weeks. 4 weeks after transplantation, tumors were weighed. No significant tumor-inhibiting activity by M-T7 was seen in (A) pancreatic cancer cell Hs766t transplanted mice (n=8, P = 0.20) or (B) breast cancer cell MDA231 transplanted mice (n=16, P= 0.24).

The growth of Hs766t and MDA231 cell lines in NOD/SCID mice was not suppressed by M-T7 treatment in our study, although there is trend toward a reduction in breast cancer cell growth, which never achieves significance (Figures 2A and 2B; P=0.24 for Hs766 and P=0.24 for MDA-231; Table 1). KG-1 tumor weights (0.28g±0.06g in saline treated group; 0.65g ±0.14g in M-T7 treated group; P=0.08) and Huh7.5 tumor (2.05g ± 0.48 in saline treated group; 1.59g ± 0.46g in M-T7 treated group; P=0.71) did not show significant difference with or without M-T7 treatment although M-T7 shows a trend toward reducing growth of KG-1 cells.

These studies suggest that blockade of the thrombolytic serine protease pathways with either Serp-1 or NSP effectively blocks pancreatic cancer cell growth *in vivo* in SCID mice while blockade of the chemokine pathways was not effective in reducing tumor growth.

### Serp-1 treatment inhibited pancreatic cancer cell proliferation *in vivo* in NOD/SCID mice but not *in vitro*

**Figure 3:**
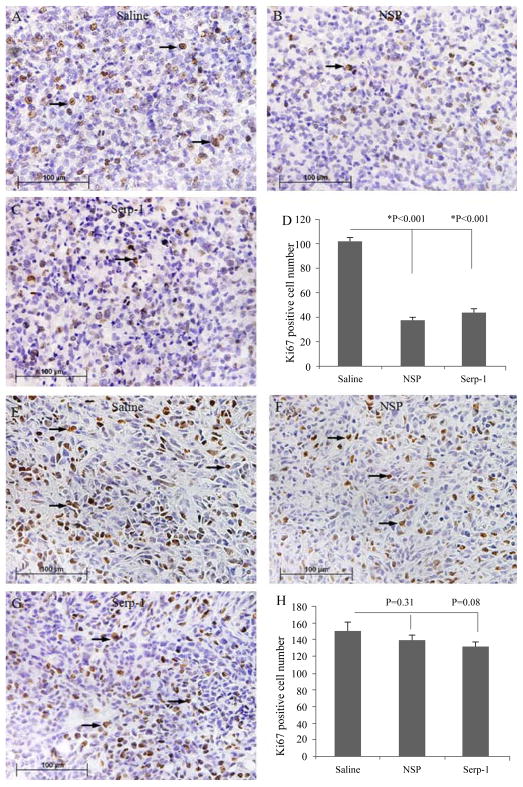
Serp-1 treatment inhibits pancreatic cancer cell proliferation *in vivo* Anti-Ki67 antibody was used to stain for proliferating cells (brown) in the tumor tissues. Pancreatic cancer Hs766t cell proliferation was inhibited in Serp-1 and neuroserpin-treated mice. (A - Saline, B- NSP, C - Serp-1) Pancreatic cancer tissues isolated from NOD/SCID mice at 4 weeks follow up and stained for Ki67 (arrows point to representative dividing cells). (D) Bar graphs illustrate comparisons of mean ± SE for positively stained cell counts for Ki67 positive cells in pancreatic cancer sections. Ki67 positive cells in five randomly selected high power fields (HPFs) were counted for each tumor tissue. The averages of positive cell counts in each treatment group were compared using ANOVA (n=40 HPFs total, *: significant level of P< 0.05). Breast cancer cell proliferation was not inhibited *in vivo* by Serp-1 and NSP treatment. (E- Saline, F - NSP, G - Serp-1) Breast cancer with Ki67 staining (arrows point to representative dividing cells). (H) Bar graphs show comparison of Ki67 positive cells in breast cancer MD-231 cell implants after treatments (n=50 HPFs total). Magnification: 400X.

To assess direct effects of each serpin on cancer cell proliferation in each treatment group, immunostaining using cell proliferation marker Ki67 was performed on tumor sections. Consistent with gross finding on tumor weights, serpin treatment decreased proliferation of pancreatic cancer cells *in vivo* as measured by Ki67 staining (P ≤ 0.001 for Serp-1 and NSP treatments; Figures 3A–3D). MDA231 breast cancer cell proliferation after implant into SCID mice was not reduced by serpin treatment (P=0.31 and 0.08, NSP and Serp-1 respectively; Figures 3E–3H).

**Figure 4:**
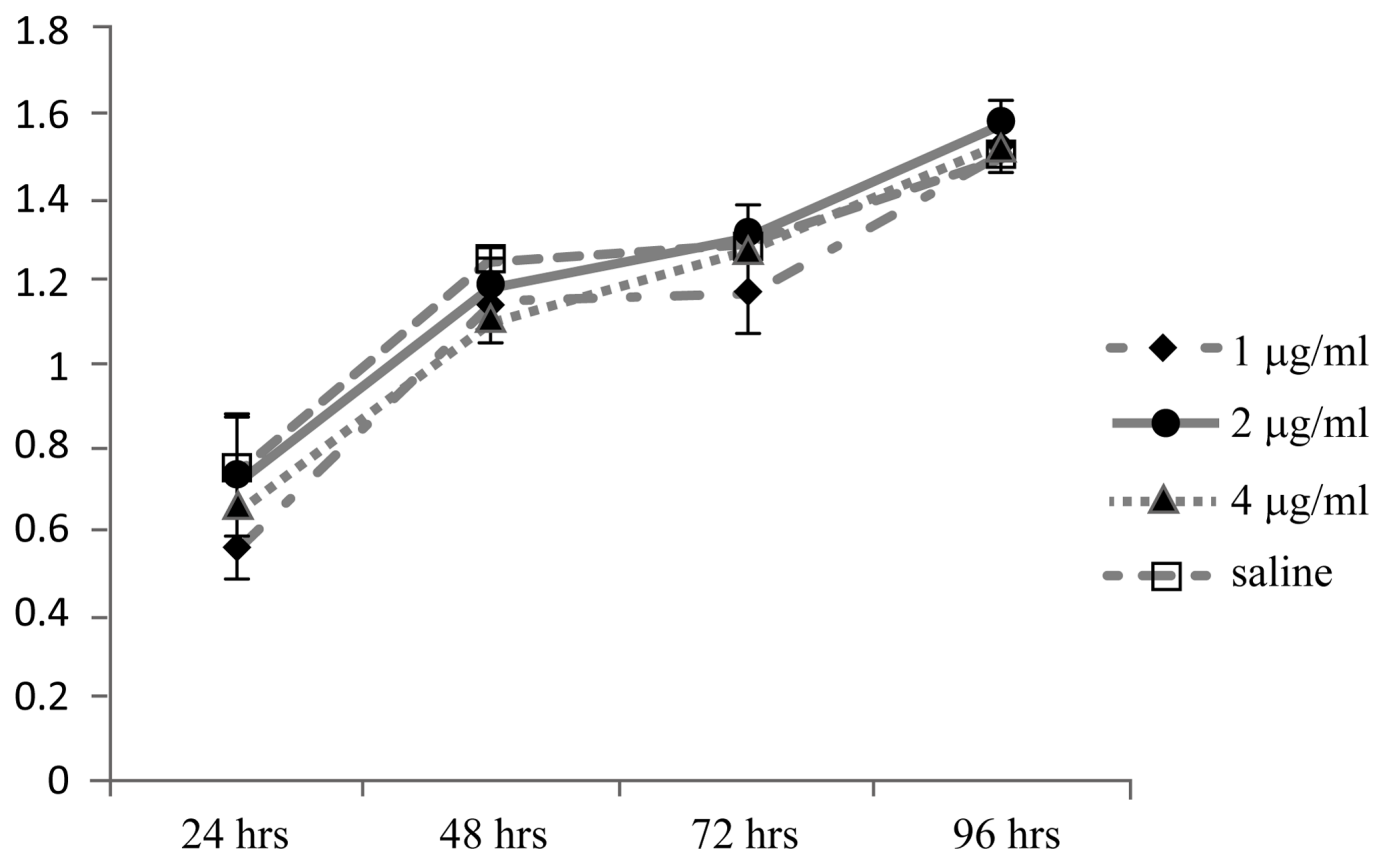
Serp-1 did not inhibit pancreatic cancer cell Hs766t growth *in vitro* Cultured Hs766t cells were treated with Serp-1 at different doses or saline. MTT assay was performed to assess cell number at 24, 48, 72 and 96 hours and the growth curves of these treatment groups were created. Cell proliferation *in vitro* for each group showed no significant difference at all time points assayed (n=3 in each time point each treatment group).

To examine for direct inhibitory effects of Serp-1 on survival or proliferation of the pancreatic cancer cell Hs766t line *in vitro* in tissue culture, cultured Hs766t cells were treated with Serp-1 for 24, 48, 72 and 96 hours. Cell proliferation was analyzed by MTT assay, and the growth curves are shown in Figure 4. No significant difference in the cell numbers were observed between Serp-1-treated group and saline-treated control group at all time points assayed, indicating that Serp-1 does not directly induce cell death nor inhibit cancer cell division in the absence of the full support of related connective tissue and inflammatory cells and factors. The cancer suppressive effects of Serp-1 observed *in vivo* thus is proposed to involve interaction of different cell types within the tumor, including cancer cells and stromal cells such as fibroblasts, vascular endothelial cells and a range of inflammatory cells.

### Serp-1 treatment decreased myeloid-derived suppressor cells in NOD/SCID mice after pancreatic cancer cell implant

**Figure 5:**
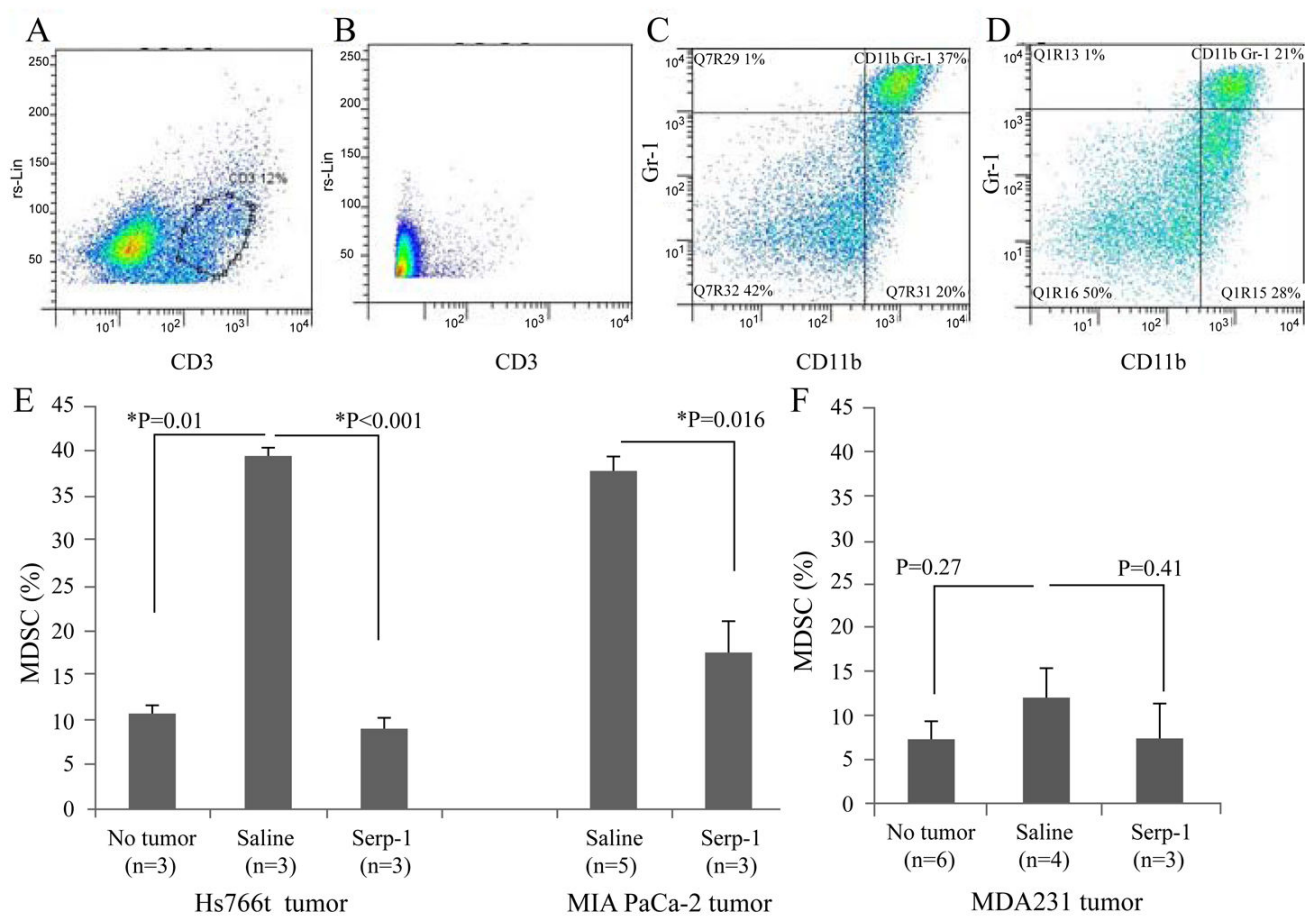
Serp-1 decreased MDSCs in NOD/SCID mice after pancreatic cancer cell transplantation (A) CD3 staining of splenocytes from a C57BL6 mouse is provided as a positive control. (B) Lack of CD3 positive T cells is shown in the NOD/SCID mice used for cancer cell transplantation. (C) Representative dot plot illustrates the MDSC population in mice with pancreatic cancer after control treatment with saline. (D) Representative dot plot illustrating MDSC population positive for CD11b and Gr-1 markers for MDSC in mice with pancreatic cancer treated with Serp-1. (E) MDSCs were increased with pancreatic cancer growth (P=0.01) and significantly decreased after Serp-1 treatment in mice transplanted with Hs766t (n=9 total, P<0.001) or MIA PaCa-2 cells (n=8 total, P=0.016). (F) MDSC cell counts assayed by flow cytometry in splenocytes from SCID mice after MD-231 breast cancer growth were not significantly altered after Serp-1 treatment (n=13 total, P=0.41).

In order to examine which inflammatory cells were potentially modified by Serp-1 treatment we examined mouse splenocytes from NOD/SCID mice with pancreatic cancer cell implants with and without Serp-1 treatments, using flow cytometry analysis. NOD/SCID mice do not have functional lymphocytes and thus lymphocytes were analyzed as a control to confirm the absence of T cell populations. The NOD/SCID mice lacked CD3 positive T lymphocytes, consistent with the reported phenotype of these mice (Figures 5A and B).

MDSC consists of a heterogeneous population of immature myeloid cells accumulating in most cancer patients and experimental cancer animal models [20]. This cell population has been found to suppress anti-tumor immunity and the increase of MDSC numbers is associated with advanced disease and poor prognosis [21]. MDSCs are induced by some cancers and by other inflammatory conditions such as infectious microorganisms and autoimmune diseases. Suppression of MDSCs is reported to inhibit tumor growth both in animal models and in studies in patients with pancreatic cancer or other cancers [5,22]. Our study reported here demonstrates inhibitory activity for Serp-1 and NSP on growth of pancreatic cancer cell implants in SCID mice. As the first step to explore the underlying mechanism, we assessed whether the inhibition of tumor growth is associated with suppression of MDSCs.

Splenocytes were isolated from NOD/SCID mice in all treatment groups and labeled with fluorescence-conjugated antibodies for flow cytometry analysis. Mouse MDSCs express both the monocytic marker CD11b and granulocytic marker Gr-1. The proportion of MDSCs was measured by gating the CD11b/Gr-1 double positive cell population within the whole population of splenocytes (Figure 5C and 5D). An increased MDSC population was seen in the saline treated control mice bearing larger pancreatic cancer implants (P ≤ 0.01, Figure 5C and 5E) when compared to the mice without tumor implants. MDSC cell counts were significantly decreased after Serp-1 treatment in tumor-bearing mice (P ≤ 0.001 for Hs766t tumors and P<0.02 for MIA PaCa-2 tumors Figure 5E).

However, in NOD/SCID mice transplanted with breast cancer MDA231 cells, the MDSC population was neither increased after tumor growth nor reduced by Serp-1 treatment (Figure 5F). The reason for the lack of MDSC response in this breast cancer model is unknown but is consistent in that this lack of activity is associated with the lack of Serp-1 efficacy on this breast cancer cell isolate.

Splenocyte subpopulations positive for CD206, a macrophage and dendritic cell marker, in each treatment group after Hs766t xenograft did not show significant differences (12.7 ± 5.9% in NSP treated group; 0.67 ± 0.67% in Serp-1 treated group; 9.3 ± 5.9% in saline treated group; P>0.26).

### Serpin treatment decreased infiltration of macrophages in pancreatic cancer implants in SCID mice

**Figure 6:**
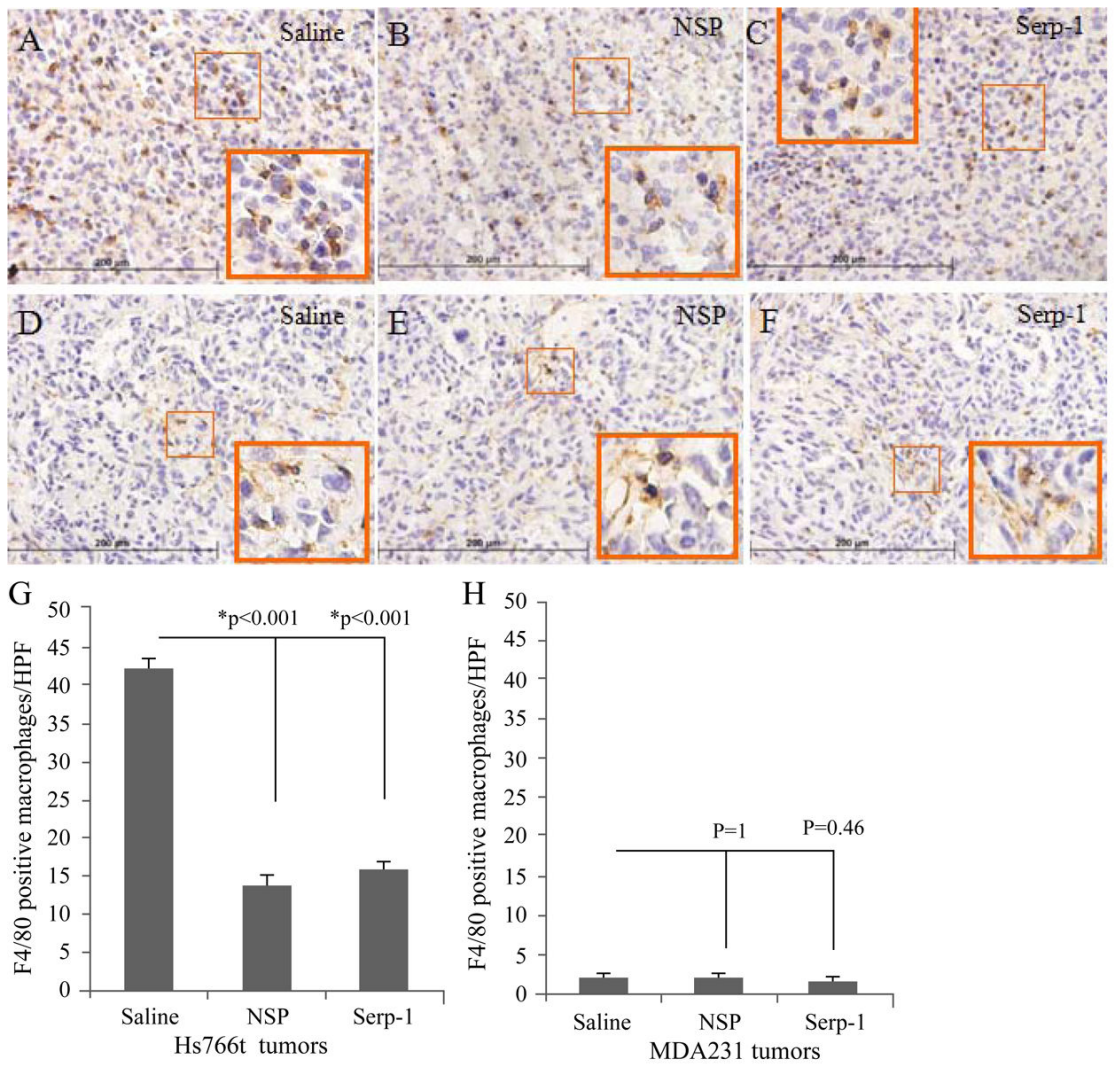
Serpin treatment decreased macrophage infiltration in pancreatic cancer xenografts Macrophages in the tumor tissue were stained with F4/80 antibody. Positive cells in five randomly selected high power fields (HPFs) were counted for each tumor section. The averages for positive cell counts from each treatment group were compared by ANOVA. (A–C) Representative tumor sections immunostained for macrophages (brown stain) in pancreatic cancer tissue are provided from saline (A), NSP (B) and Serp-1 (C) treatment groups. (D–F) Positively stained macrophage cells in breast cancer tissue from saline (D), NSP (E), and Serp-1 (F) treatment groups are also shown. (G) Positively stained macrophages in pancreatic cancer tissues were significantly decreased after serpin treatment (P<0.001, n=40 HPFs total). (H) However, serpin treatment did not significantly change macrophage number in breast cancer xenografts (P>0.4, n=35 HPFs total). Magnification 400X for histology sections and 1000X for higher power insets for each stained section * indicates significance of P ≤ 0.05.

Tumor-associated macrophages (TAMs) are an important cellular population in the tumor stroma. These cells are implicated as a part of the inflammatory microenvironment that promotes cancer related angiogenesis and cancer progression by releasing soluble mediators that support survival, proliferation and migration of vascular endothelial cells and cancer cells [23]. On immunohistochemical staining analysis, the number of positively stained macrophages in the pancreatic cancer tissue isolates was significantly decreased in NSP (Figure 6B) and Serp-1 (Figure 6C) treated mice (P<0.001, Figures 6A–C and 6G), when compared to saline treated controls. This suppression of cell counts in the pancreatic tumors suggests that Serp-1 and NSP inhibit macrophage invasion into the tumor. There were small numbers of macrophages (1~2 per 400X high power field) present in breast cancer tissues and serpin treatment did not significantly alter the number of macrophage cells detected in breast cancer tissues in our model (Figures 6D–6F and 6H). This finding suggests that this specific breast cancer model relies less on macrophages for tumor growth, a possible reason for its lack of responsiveness to serpin treatment.

### Exogenous uPA antagonizes Serp-1 inhibition of pancreatic cancer xenograft growth

**Figure 7:**
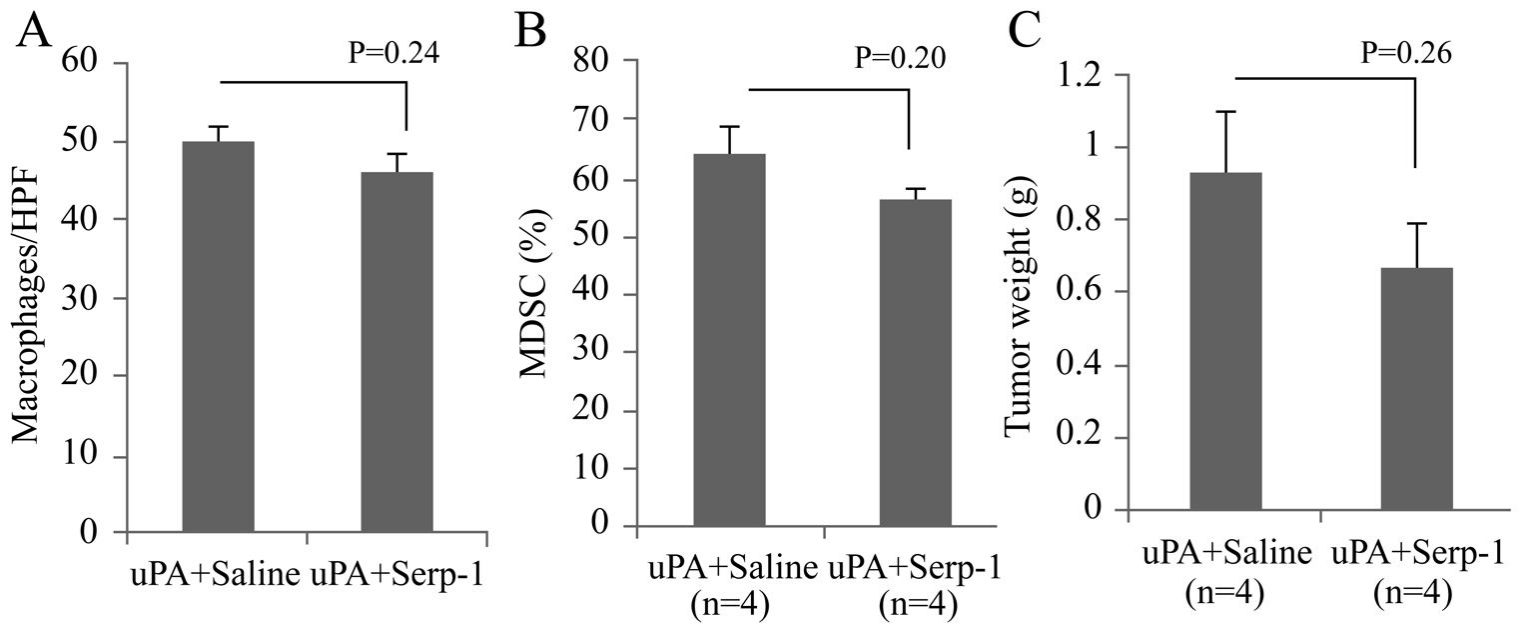
Augmentation of uPA activity through uPA injection antagonized Serp-1 inhibition of pancreatic cancer growth. Hs766t cells suspended in uPA were injected subcutaneously into NOD/SCID mice, which then received Serp-1 or saline treatment with the same dosage and duration as described for prior studies. Serp-1 treatment no longer significantly inhibited macrophage infiltration in tumors (A, P=0.24, n=20 HPFs per group) or decreased MDSCs in the spleens (B, P=0.20, n=4 per group). (C)Tumor growth in these mice was not suppressed by Serp-1 treatment (P=0.26, n=4 per group).

Serp-1 has been demonstrated to be an effective inhibitor of uPA and to function as a suppressor of inflammation [8–10]. To investigate whether the anti-inflammatory activity of Serp-1 observed in our pancreatic cancer model is due to uPA inhibition, exogenous uPA was injected together with Hs766t cells to create xenografts with increased levels of uPA. With the addition of uPA to the tumor implants, Serp-1 treatment no longer significantly decreased macrophage infiltration and MDSC mobilization (Figure 7A and 7B, P= NS), nor inhibited tumor growth in these mice (Figure 7C, P=NS), although there was a trend toward a reduction. This finding supports our hypothesis that Serp-1 mediated anti-tumor activity is produced in part through blockade of uPA in our pancreatic cancer model.

## Discussion

The use of anti-inflammatory drugs in cancer treatment is under investigation in both pre-clinical studies and clinical trials. For example, Celecoxib, a non-steroidal anti-inflammatory drug that selectively inhibits cyclooxygenase 2 (COX-2), has been evaluated in a number of clinical trials for cancer therapy [24–26]. Our current study is the first to explore the potential use of myxomavirus-derived antiinflammatory proteins in cancer therapy. This study demonstrated that myxomaviral protein Serp-1 inhibits growth of two different pancreatic cancer cell lines in NOD/SCID mice, providing a proof-of-concept that viral anti-inflammatory protein Serp-1 has the potential to be used for therapeutic application in treatment of pancreatic cancer. The use of myxomaviral chemokine-modulating protein M-T7 did not show inhibitory effect on the pancreatic and breast cancer models adopted in our present study. This lack of efficacy might reflect the complexity and redundancy of chemokine system in cancer microenvironment. Interestingly, myxomavirus itself has marked oncolytic activity on multiple pancreatic cancer cell line and *in vivo* animal models [27]. Although the mechanism remains to be elucidated, it is possible that Serp-1 play a role in the process of oncolysis.

Our study also demonstrated that neuroserpin, a mammalian serpin, has similar inhibitory effects on the growth of a pancreatic cancer cell xenograft implant. Serp-1 and neuroserpin both share the capacity to inhibit uPA and tPA, and thus we postulate that the antiinflammatory/anti-tumor activity of these two proteins is secondary to inhibition of the uPA/uPA receptor (uPAR) in immune cells and/or pancreatic cancer cells. The uPA/uPAR system has been reported to play an important role in development and progression of numerous human cancers including pancreatic cancer [6,28]. Suppression of this system impairs proliferation, migration and angiogenesis of pancreatic cancer [29,30].

Activated uPA is believed to facilitate cancer cell invasion and metastasis as well as immune cell migration by sequentially activating plasmin and matrix metalloproteinases (MMPs), which break down matrix barriers and expose adhesion sites for migrating cells. Activated uPA/uPAR and downstream proteinases also regulate the function of ECM-bound growth factors such as TGF-β, vascular endothelial growth factor (VEGF), hepatocyte growth factor (HGF), basic fibroblast growth factor (bFGF) and other growth factors implicated in cancer cell growth and angiogenesis [31].

In addition to mediating localized proteolysis of ECM components, binding of uPA with uPAR regulates cell signaling through direct interaction with integrins and other co-receptors on the cell surface, and through this mechanism may further promote cell adhesion, proliferation and migration of immune cells and cancer cells [32,33], a possible mechanism for the cancer-promoting activity of uPA/uPAR system. However, the signaling function of uPA may not depend fully upon its protease activity, but rather interactions with adjacent elements of the uPAR raft and interactions with the serpins together with uPA and the receptors on the cell surface. Serp-1 acts as a suicidal substrate for uPA [8] and thus may not necessarily directly interrupt uPA signaling or may interrupt cell activation, invasion or even signaling to different degrees in cancer cells or in inflammatory response cells that support cancer cell growth and invasion. Thus, Serp-1 may not directly inhibit cancer cell proliferation. This is consistent with our observation that Serp-1 fails to inhibit pancreatic cancer cell proliferation *in vitro* (Figure 4), but does block cancer growth *in vivo*. Although some breast cancers are known to be associated with uPA and uPAR levels, the role of uPA/uPAR in inflammatory cells associated with breast cancer may differ from interaction with pancreatic cancer cells. Serp-1 may not inhibit breast cancer growth even with a significant level of uPA expression in the cancer cells themselves, if there is no associated increase in expression of uPA or uPAR in TAM or MDSC. Furthermore, breast cancer-bearing mice in this study had no significant reduction in spleen MDSC numbers after serpin treatment with additionally less macrophage infiltration in the tumors. It is therefore likely that the breast cancer model we used in this study relies less on macrophage-mediated inflammation than the pancreatic cancer cells examined here.

Given that inflammatory cells play an important role in pancreatic cancer progression [34] and that Serp-1 lacks inhibitory activity for pancreatic cancer cell proliferation in culture, it is reasonable to postulate that Serp-1 is targeting tumor promoting innate immune responses to exert the observed anti-tumor activity *in vivo*, most likely by impeding uPA-dependent migration of inflammatory cells such as TAMs and MDSCs.

Since uPA and uPAR are expressed in a variety of immune cells, and are implicated in innate and adaptive immunity through modulating immune cell adhesion and migration, inhibiting the activity of uPA with Serp-1 and neuroserpin may reshape the inflammatory reaction in the tumors partially through inhibiting TAM infiltration as shown in this study (Figure 6). Cross-talk between MDSCs and macrophages has the potential to convert macrophages to a M2 phenotype which promotes tumor progression [35]. As uPA/uPAR function has been correlated with mobilization of MDSC from the bone marrow [36,37], inhibition of uPA by Serp-1 has the potential to directly suppress mobilization of MDSC and through modulating functions of other inflammatory cells, thus favor the establishment of anti-tumorigenic microenvironment.

As the first step to evaluate the role of uPA in the anti-tumor function of serpins, pancreatic cancer Hs766t was treated with uPA and Serp-1. Exogenous uPA clearly blocked the anti-inflammation/antitumor function of Serp-1 in this cancer model (Figure 7), supporting the hypothesis that Serp-1 exerts anti-tumor function by inhibiting uPA activity. Future study aimed to characterize the functional status of inflammatory cells including MDSCs and TAMs, as well as to assess the function of uPA/uPAR in these tumors might shed more lights on the mechanism of the anti-cancer activity of Serp-1 and neuroserpin.

## Figures and Tables

**Table 1:** Numbers of NOD/SCID mice tested for each cancer cell implant and for each treatment group.

	Saline	Serp-1	Neuroserpin	M-T7
Hs766t transplanted	16	9*	6*	4
MIA PaCa-2 transplanted	5	5*	0	0
MDA231 transplanted	13	6	5	8
Huh7.5 transplanted	3	0	0	3
KG-1 transplanted	3	0	0	4

* indicates reduced tumor growth in these treatment groups.
